# Ordered mesoporous silica prepared by quiescent interfacial growth method - effects of reaction chemistry

**DOI:** 10.1186/1556-276X-8-484

**Published:** 2013-11-16

**Authors:** Hatem M Alsyouri, Malyuba A Abu-Daabes, Ayah Alassali, Jerry YS Lin

**Affiliations:** 1 Department of Chemical Engineering, The University of Jordan, Amman 11942, Jordan; 2 Department of Pharmaceutical and Chemical Engineering, German-Jordanian University, Amman 11180, Jordan; 3 School of Engineering for Matter, Transport and Energy, Arizona State University, Tempe AZ 85287, USA

**Keywords:** Self assembly, Quiescent growth, Interfacial synthesis, Ordered mesoporous silica, Synthesis-structure relationship

## Abstract

Acidic interfacial growth can provide a number of industrially important mesoporous silica morphologies including fibers, spheres, and other rich shapes. Studying the reaction chemistry under quiescent (no mixing) conditions is important for understanding and for the production of the desired shapes. The focus of this work is to understand the effect of a number of previously untested conditions: acid type (HCl, HNO_3_, and H_2_SO_4_), acid content, silica precursor type (TBOS and TEOS), and surfactant type (CTAB, Tween 20, and Tween 80) on the shape and structure of products formed under quiescent two-phase interfacial configuration. Results show that the quiescent growth is typically slow due to the absence of mixing. The whole process of product formation and pore structuring becomes limited by the slow interfacial diffusion of silica source. TBOS-CTAB-HCl was the typical combination to produce fibers with high order in the interfacial region. The use of other acids (HNO_3_ and H_2_SO_4_), a less hydrophobic silica source (TEOS), and/or a neutral surfactant (Tweens) facilitate diffusion and homogenous supply of silica source into the bulk phase and give spheres and gyroids with low mesoporous order. The results suggest two distinct regions for silica growth (interfacial region and bulk region) in which the rate of solvent evaporation and local concentration affect the speed and dimension of growth. A combined mechanism for the interfacial bulk growth of mesoporous silica under quiescent conditions is proposed.

## Background

Discovery of the surfactant-based supramacromolecular templating assembly over the past two decades added new dimensions for material synthesis with tuned properties. A wide range of periodic porous materials with controlled structures and morphologies including the M41S [1] and SBA-n [2,3] silica families, MSU-n systems [4,5], aluminosilicates [6], metal oxides [7], PMO organosilicas [8,9], hybrid nanocomposites [10], and carbon materials [11] has been developed. Extensive variations of the reaction conditions such as surfactant type, mixed surfactants, silica source, mixed inorganic sources, counterion, (co)solvent, pH adjustment, shearing stress, temperature, and many other parameters have contributed to comprehensive understanding of the mechanism of formation. Accordingly, several pathways were proposed to describe the mechanism of mesophase formation (e.g. S^+^I^−^, S^−^I^+^, S^0^I^0^, S^+^X^−^I^+^, S^0^I^0^, and S^0^H^+^X^−^I^+^) which enabled the precise manipulation of product properties [12].

Acid synthesis through the S^+^X^−^I^+^ pathway is one of the important developments of mesoporous materials. It can generate a number of industrially important morphologies [13,14] due to the weak interaction between similarly charged cationic silica precursor (I+) and cationic surfactant (S^+^) mediated by the anionic counterion (X^−^) supplied by an acid or salt. The weak interaction triggers several topological defects that emerge as rich morphologies such as spheres, rods, fibers, and gyroids [15,16]. Control over the S^+^X^−^I^+^ acidic interaction was broadly investigated to induce structural transformation and to tune the morphological features. This was done by varying the type of surfactant and co-surfactant [17] or co-solvent [18] (influence S^+^), type and concentration of acid [19] or salt [20] (affect X^−^), as well as pH [21] and silica type [22] (affect I^+^). Shear forces induced by mixing also play a vital role in determining the final morphology of the product [23].

Synthesis of mesoporous materials without stirring is a unique approach for interacting reagents under stagnant conditions. It was used to produce interesting morphologies of well-defined geometries within the bulk [24] or at oil–water interface [25] of the growth medium. It is worthy here to distinguish between ‘quiescent’ and ‘static’ conditions because literature may refer to them interchangeably although they are fundamentally different. The distinct feature lies in mixing while adding the silica source to the surfactant solution. In quiescent conditions, a silica precursor is added without mixing it to a premixed water phase containing the surfactant, while in static conditions, a silica precursor is mixed well with the water phase before holding the solution static. Therefore, upon aging, the silica species are available homogenously all over the solution in the static growth medium and thus grow in the bulk, while they have to diffuse across an interface in quiescent conditions and grow in the interface and/or the bulk regions. The growth time in both cases is remarkably longer (days) than mixed conditions (minutes to hours), but it is obviously longer under quiescent conditions due to diffusion limitations.

Acidic syntheses under both static and quiescent conditions were demonstrated to grow regular morphologies such rods, fibers, films, and spheres [16,26-30]. Moreover, the slow growth under static conditions allowed better tracking and understanding of the mesostructure and morphology formation mechanism [22,31]. The quiescent growth, which was handled to a lesser extent, introduces a stable interface between the silica and water phases, the stability of which depends on the partial miscibility between hydrophobic silica source and hydrophilic water phase. We will refer to this interaction mode as quiescent interfacial growth, and it will be the focus of this work. Stucky and coworkers have used this approach to grow a number of interesting morphologies at the silica-water interface including the ordered mesoporous silica fibers which has a unique helical pore structure [32].

Since the first report on mesoporous silica fiber [32], most of the subsequent quiescent interfacial studies were focused on the fibers and their characteristics, e.g., pore orientation [33-35], formation kinetics [36,37], and diffusional properties [38-40]. Little attention was given to investigate the quiescent interfacial method itself and the physical chemistry involved in a comprehensive manner compared to the well-studied mixed and static systems. This technique is differentiated by the way silica precursor is administered and thus has unique features of reaction and morphological evolution. Besides, this technique can be utilized to overcome challenges associated with pore orientation in membrane synthesis. For example, we have extended the quiescent interfacial method to fabricate inorganic membranes with favorable pore orientation by a new approach called counter diffusion self-assembly [41,42].

The purpose of the present work is to shed some light on the formation of mesoporous silica under quiescent conditions by covering a wide range of previously untested variables. More specifically, by starting from the fiber producing conditions, we will examine the influence of acid type and content (HCl, HNO_3_, and H_2_SO_4_), silica precursor type and hydrophobicity (tetrabutyl orthosilicate (TBOS) and tetraethyl orthosilicate (TEOS)), and surfactant type (ionic: cyteltrimethlammonium bromide (CTAB); and nonionic: Tween 20 and Tween 80) on the product type and structural properties. Most of these variables, except the second one [36], are being tested for the first time. Mesoporous silica products have been grown quiescently for a sufficient period of time and were then tested by nitrogen porosimetry, electron microscopy, and X-ray diffraction (XRD) to characterize the morphology. These results were used to understand general features of the quiescent interfacial method and its products.

## Methods

### Materials

TEOS (Si(OCH_2_CH_3_)_4_, 98%) and TBOS (Si(CH_3_CH_2_CH_2_CH_2_O)_4_, 97%) obtained from Sigma-Aldrich (St. Louis, MO, USA) were used as silica sources. Three surfactants were employed: CTAB (from Sigma Aldrich) cationic surfactant and two poly(ethylene oxide) (PEO)-based nonionic surfactants, PEO sorbitan monolaurate (known as Tween 20, from GCC, UK) and PEO sorbitan monooleate (known as Tween 80, from VWR, USA). Analytical grade hydrochloric (37%) and nitric (65%) acids were diluted to 6 M for experimental use. All dilutions and reactions were undertaken using deionized water.

### Synthesis

**Figure 1:**
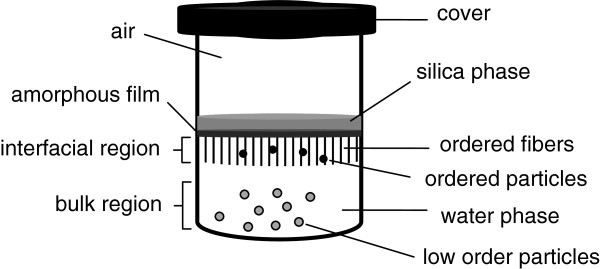
A schematic of the quiescent interfacial growth method in a beaker.

A summary of samples and growth variables of this work is given in Table 1. Mesoporous silica fiber (MSF) sample that yields ordered mesoporous silica fibers will be used as a reference for comparison of variable outputs. Starting from the MSF molar recipe (100 H_2_O/3.34 HCl/0.026 CTAB/0.05 TBOS), other samples were pursued by exchanging the corresponding variable. Samples MS7 and MS12 comprise multiple runs prepared under a range of acid molar ratios: 0.2 to 3.34 nitric acid and 1.0 to 3.34 sulfuric acid, respectively. The low-acid content of samples MS7 and MS12 was reported earlier but was not fully interpreted [43]. These results were added to this paper to provide a comprehensive analysis. The quiescent interfacial growth of mesoporous silica in a beaker is illustrated in Figure 1. The water phase is a hydrophilic mixture containing deionized water, surfactant, and acid catalyst, while the silica phase consists of the silica precursor which is generally hydrophobic to slow down its diffusion into the water phase.

**Table 1:** A summary of samples and molar ratios per 100 mol of water

**Sample**	**Acid**			**Surfactant**			**Silica source**	
	**HA**	**NA**	**SA**	**CTAB**	**T20**	**T80**	**TBOS**	**TEOS**
MSF	3.34			0.026			0.05	
MS-7		0.20 to 3.34		0.026			0.05	
MS-12			1.00 to 3.34	0.026			0.05	
MS-4	3.34			0.026				0.08
MS-6b		3.41		0.026				0.08
MS-5a	3.34				0.01			0.05
MS-5b	3.34					0.01		0.05

All preparations took place at room temperature and quiescent (no-mixing) conditions. HA, hydrochloric acid (HCl); NA, nitric acid (HNO_3_); SA, sulfuric acid (H_2_SO_4_); T20, Tween 20; T80, Tween 80.

In a typical experiment, water phase is prepared by mixing the surfactant, water, and acid at room temperature until a clear solution is obtained. The mixing is stopped, then silica source is added slowly as a thin layer standing on top of the water phase. The beaker is aged in quiescent (stagnant) conditions for a desired period of time. This type of growth is generally slow and would take over 2 days to produce silica particles and can extend to 14 days in some cases. Silica growth initiates at the water-silica interface as an amorphous layer, then it proceeds inside the water phase as shown in Figure 1 yielding mesoporous silica with a variable degree of order (fibers are more ordered than particulates). At the end of the growth, silica product is collected, dried, and calcined at 560°C for 6 h at heating and cooling rates of 1°C/min.

### Characterization

Nitrogen physisorption isotherms were measured using PMI and Micromeritics ASAP-2020 (Norcross, GA, USA) automated sorptometers at liquid nitrogen temperature (77 K) after outgassing under vacuum at 200°C (473 K) for at least 3 h. Surface area was calculated by applying the Brunauer-Emmett-Teller (BET) theory to the adsorption isotherms over a relative pressure (p/p_o_) range of 0.10 to 0.30. The total pore volumes were evaluated from the adsorption isotherm using the single-point method at a relative pressure of 0.995. Average pore diameter was calculated using the Barret-Joyner-Halenda (BJH) model from the desorption isotherm.

The powder XRD patterns were measured on a Philips X’pert Pro XRD instrument (X’Pert, PANalytical B.V., Almelo, The Netherlands) operating with Cu-Kα_1_ radiation (*λ* = 1.54055 Å) at 40 kV using a Ni filter to remove the Cu-Kβ line. Data points were recorded using a spinner system with a 0.25-in. slit mask between 2*θ* angles of 1.5° to 8° with a step size of 0.017° and a scan speed of 15 s per step. Scanning electron microscopy (SEM) images were recorded on a REM JEOL 5900 LV microscope (JEOL Ltd., Akishima, Tokyo, Japan) operating at 25 kV with a resolution of 5 nm and a nominal magnification of 3.0 × 10^6^. For SEM, the powdered samples were used without any pretreatment or coating. Transmission electron microscopy (TEM) was measured on a JEOL-2011 electron microscope operating at 200 kV. Prior to the measurements, the samples were suspended in ethanol solution and dried on a copper-carbon grid.

## Results and discussion

### Mesoporous silica fibers

We have investigated the MSF in a number of earlier publications and reported their microstructural [37] and diffusional properties [38,40]. In this work, part of these results will be presented as a reference to delineate effects of other variables. The growth starts with a thin amorphous layer at the interface of the two-phase mixture within 2 days of induction followed by slow growth of a white matrix of fibers attached to the thin amorphous layer in the water phase. Regular particulates also emerge along the fibers in the water bulk and precipitate at the bottom of the beaker (see Figure 1). We noticed that 10 to 14 days is a typical period for fiber growth over which the yield and pore order of fibers increase markedly with time. The long time is due to quiescent conditions where species has to interdiffuse slowly in absence of any bulk movement. TBOS species diffuse from the silica layer into the water phase; surfactant micelles also diffuse in the water bulk to interact with silica species in the interfacial region. Water and alcohol (resulting from the hydrolysis) diffuse as well and evaporate at the interface. This was reported to influence the growth in this method [42].

**Figure 2:**
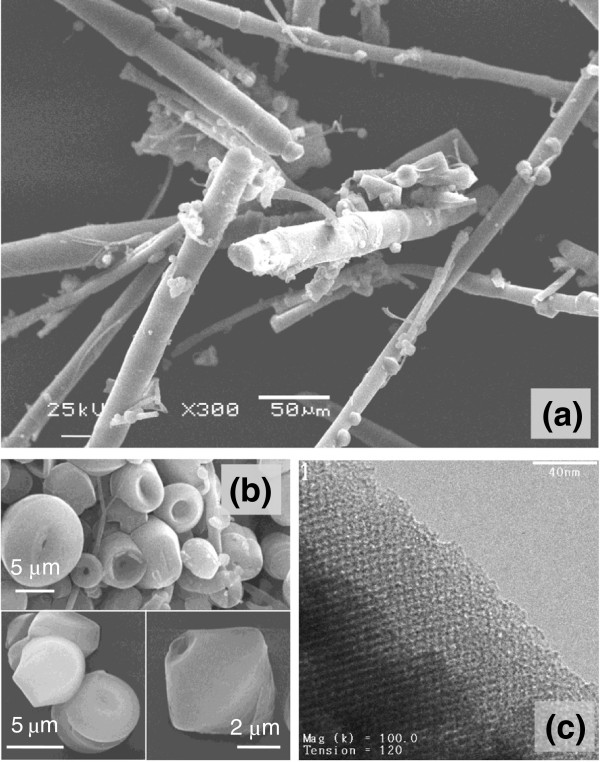
**Electron micrographs of MSF sample. (a)** SEM of fiber morphology, **(b)** SEM of some co-existing morphologies, and **(c)** TEM of fibers.

**Figure 3:**
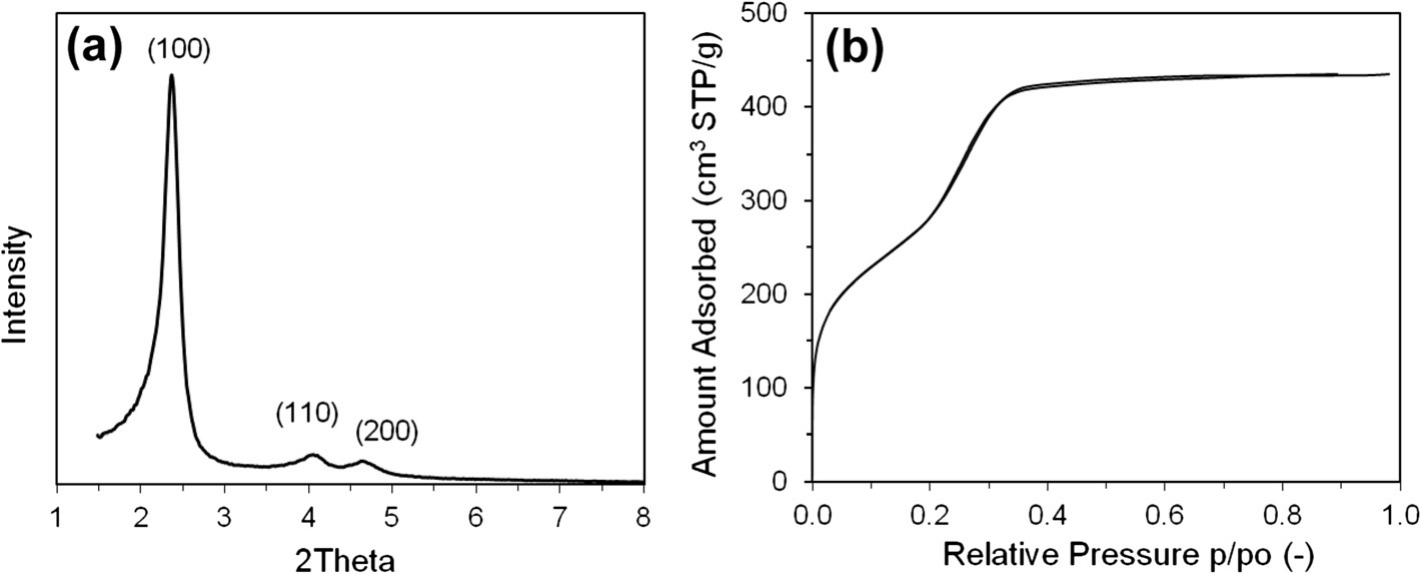
**XRD pattern (a) and N**_**2 **_**ads**/**desorption isotherms (b) of mesoporous silica fibers.**

SEM images in Figure 2 illustrate the typical fiber and co-existing particulate morphologies. The fibers can grow to a length scale of millimeters, but they break easily yielding average dimensions of 500-μm length × 25-μm diameter. Gyroids are examples of co-existing particulates having comparable diameters to fibers. They apparently start to grow within the water phase and precipitate when they become denser than the aqueous solution. A TEM image (Figure 2c) depicts the ordered pore structure of the fibers, which corresponds to a 2D hexagonal mesostructure of p6mm symmetry. The ordered pores extend along the fiber axis in a helical or circular fashion as revealed by microscopy [39] and diffusional investigations [38,40]. Such architecture is interesting in catalysis and controlled release applications. Ordered pore structure was further confirmed by XRD (Figure 3a). The pattern displays a high intensity primary reflection at 2.37° of *d* spacing = 3.72 nm which confirms the hexagonal structure. Two additional secondary reflections are also observed verifying a long range order. The peaks appear in the low range of 2*θ* between 1.5° to 6° and are indexed as (100), (110), and (200) planes.

N_2_ sorption isotherms of MSF measured at 77 F are shown in Figure 3b. They have type IV responses typical to mesoporous materials with well-defined capillary condensation step at 0.3 p/p_o_ that is absent of any hysteresis. This indicates a uniform and narrow pore size distribution. Textural properties obtained from the XRD patterns (*d* spacing and lattice parameter *a*_0_) and sorption isotherms (average pore size, surface area, and pore volume) for all samples are summarized in Table 2. The fibers have a BET surface area of 1,008 m^2^/g and a total pore volume of 0.64 cm^3^/g. The pore size, calculated from the desorption isotherm using the BJH theory was found to be 2.35 nm with a uniform distribution. It is noteworthy that the BJH theory underestimates the pore size. A more reliable model such as the density functional theory yields a pore size of 3.53 nm for our sample [40]. However, due to the simplicity of the BJH method, BJH values were used for contrasting pore sizes among different samples.

**Table 2:** Structural properties of the mesoporous silica products

**Sample**	***d***_**100 **_**spacing**^**a **^**(nm)**	***a***_**0**_^**b **^**(nm)**	***w***_**BJH**_^**c **^**(nm)**	**Wall thickness**^**d **^**(nm)**	***S***_**BET **_**(m**^**2**^**/g)**	***V***_**tot**_^**f **^**(cm**^**3**^/**g)**
MSF	3.72	4.3	2.35	1.95	1,008	0.64
MS7						
0.2 NA	4.60	5.31	3.01	2.30	624	0.43
0.5 NA	4.70	5.42	2.97	2.45	560	0.40
1 NA	3.42	3.95	2.5, 3.8^i^	0.92	1,454	1.26
2 NA	3.20	3.69	2.90	0.30	799	0.62
3.34 NA	4.34	5.01	2.86	1.48	887	0.54
MS12						
1 SA	3.27	3.78	2.49	0.78	1,506	0.98
2 SA	3.42	3.95	2.56	0.86	1,504	0.96
3.34 SA^g^						
MS4	3.64 to 7.21	4.3 to 8.3	3.70	1.73 (1.91^e^)	475	0.28
MS6b	4.10	4.73	2.64	1.46	299	0.16
MS5a	^h^	^h^	3.00	-	375	0.24
MS5b	6.15	7.10	3.70	2.45 (2.85^e^)	199	0.17

^a^Calculated from 2*θ* value corresponding to the (100) peak in the XRD pattern using Braggs law; ^b^$a_{0}=d_{100}\times \sqrt{\left(4/3\right)}$; ^c^BJH pore diameter calculated from the desorption isotherm; ^d^For poorly ordered materials wall thickness = *d*_100_ − *w*_BJH_, the better order samples (MSF, 0.1 NA and 0.2 NA) are calculated as *a*_0_ − *w*_BJH_; ^e^Estimated from TEM images; ^f^Single-point total pore volume at p/p_o_ = 0.995; ^g^No growth was observed with this molar value of sulfuric acid over the growth period; ^h^Not determined from XRD graph; ^i^Bimodal pore size distribution.

### Effects of acid type and counterion

The effect of acid and associated counterion is represented by group MS7 using nitric acid (NO_3_^−^ monovalent counterion) and group MS12 using sulfuric acid (SO_4_^2−^ divalent counterion). Acid content was varied in the range of 0.2 to 3.34 mol HNO_3_ and 1 to 3.34 mol H_2_SO_4_ in the respective groups per 100 mol H_2_O. Both acids displayed a noteworthy influence on the product structure and morphology. Growth sequence exhibited a turbid solution in the water phase within 2 days; with time, this turbidity develops in the water bulk into a white soft precipitate. According to visual observations, the rate of formation was faster for nitric acid and proportional to the acid content. However, for sulfuric acid at a high concentration (3.34 SA), no product was formed over the entire growth period (14 days) indicating a hindered or slow growth.

**Figure 4:**
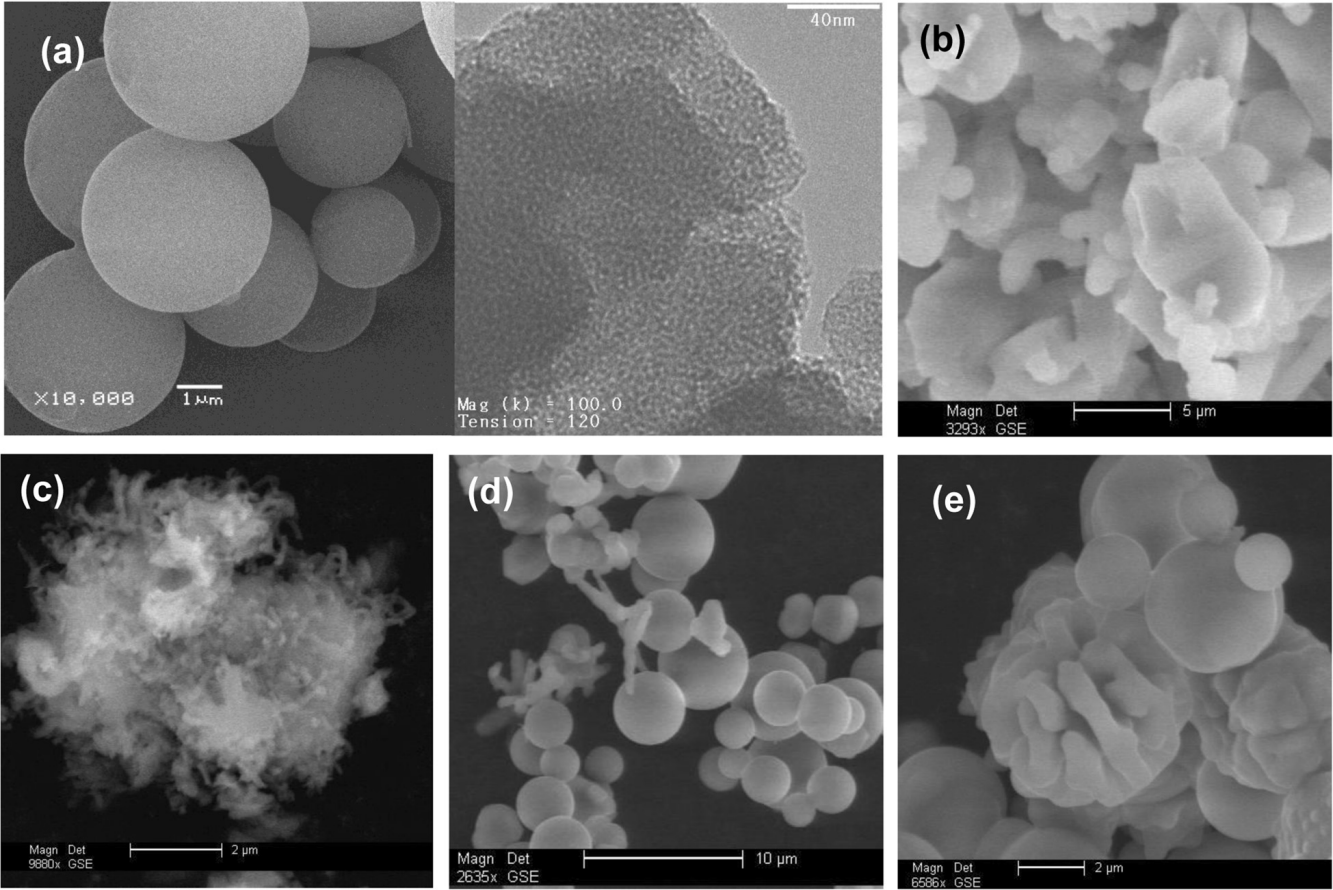
**SEM images of sample MS7 at different nitric acid contents. (a)** 3.34, **(b)** 2.0, **(c)** 1.0, **(d)** 0.5, and **(e)** 0.2 mol relative to 100 mol water. Image **(a)** contains the corresponding TEM image.

**Figure 5:**
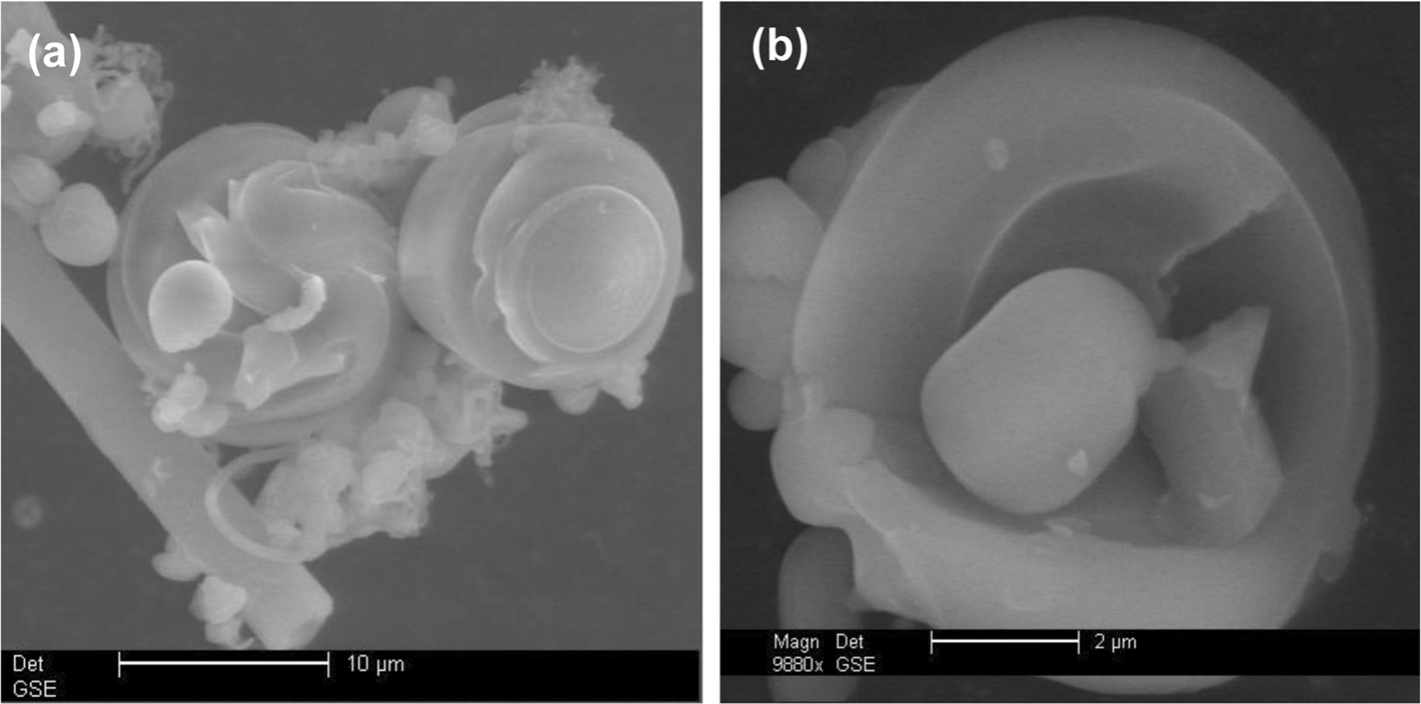
**SEM images of sample MS12 at different sulfuric acid contents. (a)** 1.0 and **(b)** 2.0 mol relative to 100 mol water. No growth was observed with the 3.34 molar ratio.

Unlike HCl, synthesis with HNO_3_ or H_2_SO_4_ displays nonfibrous products. Fibers were not seen as a distinctive output at any condition undertaken with these acids. As shown in Figure 4a, at 3.34 nitric acid molar content (sample 3.34 NA), the equivalent sample to MSF, spheres with smooth texture were observed as the dominant shape having a size distribution of less than 10 μm. This shape disappeared at intermediate ratios (2 NA and 1 NA) where only disordered loose particles and films were seen (Figure 4b,c), whereas at lower contents (0.5 and 0.2), the spheres were observed again but with corrugated surfaces along with other random particulates as shown in Figure 4d,e. Apparently, nitric acid content influenced the morphology, giving spheres as the prevailing output. No correlation was observed between the acid content and sphere size, but it apparently affected the rate of condensation and thus the spherical texture. When employing sulfuric acid (SA), multishapes were seen both at 1 SA and 2 SA (see Figure 5). Regardless of the content, a nonuniform mix of shapes was obtained including spheres (solid and hollow), small fibers, and whirling rods. At a higher molar ratio (3.34 SA), no product was obtained, suggesting that at high sulfuric acid ratios, the growth becomes extremely slow.

**Figure 6:**
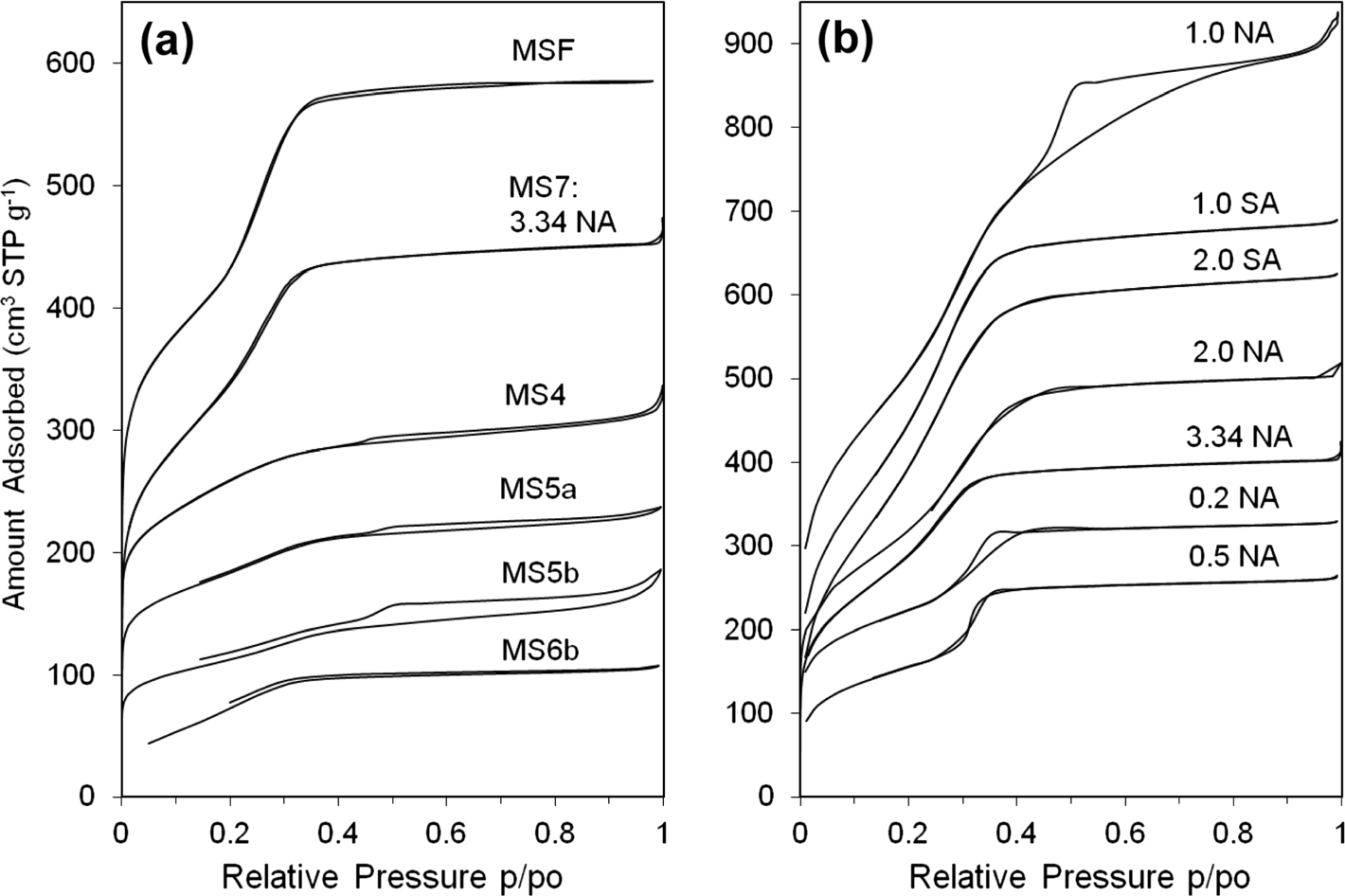
**Nitrogen adsorption**-**desorption isotherms of mesoporous silica prepared under quiescent interfacial growth method. (a)** All samples and **(b)** samples MS7 and MS12 prepared using various molar ratios of nitric acid (NA) and sulfuric acid (SA), respectively. Some isotherms were shifted upwards for proper comparison.

**Figure 7:**
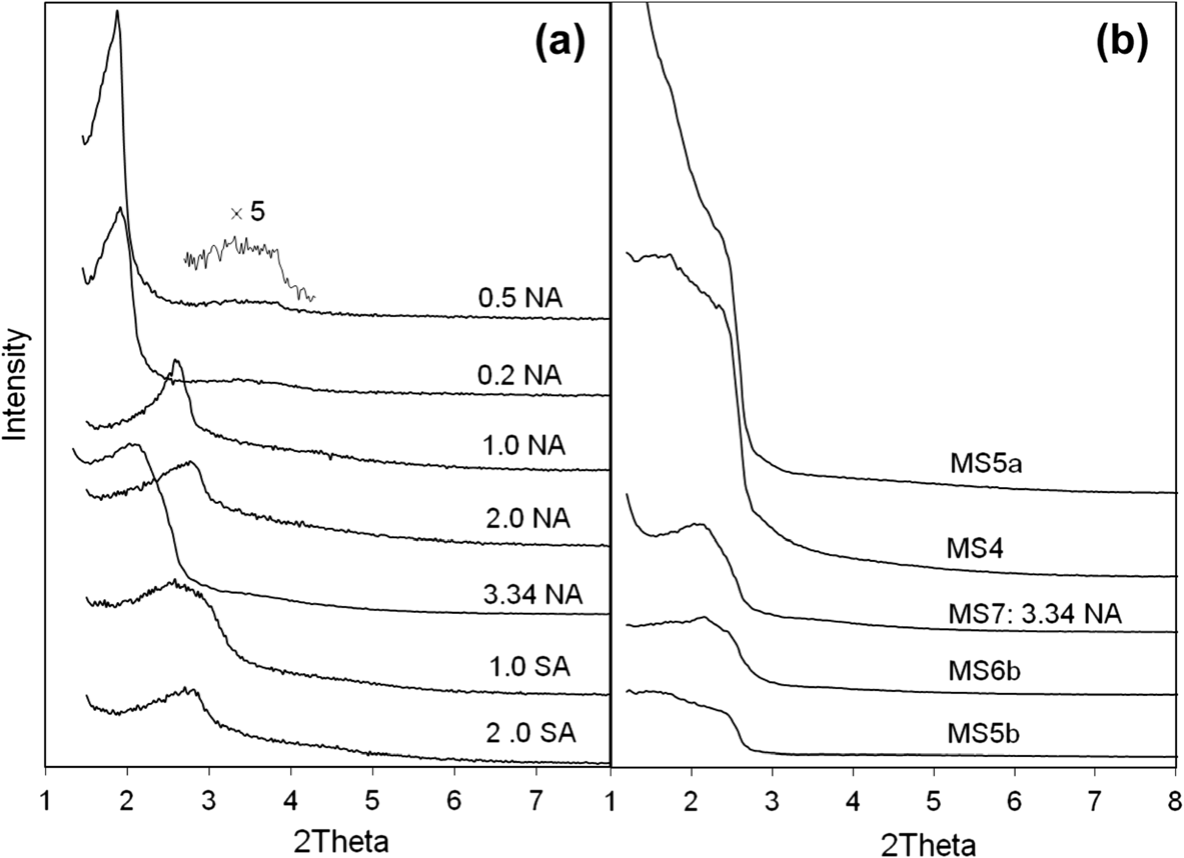
**XRD patterns of mesoporous silica products. (a)** Samples MS7 and MS12 prepared at different molar ratios of nitric acid (NA) and sulfuric acid (SA) respectively and **(b)** all remaining samples. Sample MS12 at 3.34 SA is not shown because no product was grown throughout the growth period.

Microstructural properties studied by XRD and N_2_ sorption isotherms were collectively presented for all samples in Figure 6 (sorption isotherms) and Figure 7 (XRD patterns) to clarify differences associated with each condition. These data were used to calculate the pore structural properties presented in Table 2. First, we will talk about the sample prepared at 3.34 NA which is the mutual counterpart of the silica fiber sample prepared using HCl; we will then discuss the effect of varying the acid content for both nitric and sulfuric acids.

As shown in Figure 6a, the sorption isotherms of the spherical silica precipitated at 3.34 NA M are very comparable to those of the fibers. The isotherms have type IV mesoporous isotherms showing capillary condensation step at p/p_o_ ~ 0.3 that is absent of any hysteresis. The relatively steep capillary condensation indicates a uniform size distribution with a pore diameter of 2.86 nm (compared to 2.35 nm of MSF) and respective surface area and pore size of 887 m^2^/g and 0.54 m^3^/g. The fibers and spherical particles possess comparable pore area properties except that the nitric acid causes a little swelling to the pore size. The pore order of the 3.34 NA sample is reflected in the XRD pattern in Figure 7a. The pattern shows one broad (100) peak, indicating a low degree of pore arrangement. The low order of pores in spheres is verified by TEM in Figure 4a which reveals wormlike mesoporous channels. It is visible that substituting HCl with an equivalent amount of HNO_3_ yields a spherical product with uniform mesoporous channels but causes the loss of pore order.

Progressive decrease in the molar ratio of NA causes notable changes in the morphology and microstructure of the product. At intermediate molar ratios (1 NA and 2 NA), loose fine particulate and film products were formed with a disordered structure. Their XRD patterns in Figure 7a show only a one broad (100) reflection shifted to a slightly higher angle than sample 3.34 NA (the high order small peaks are not discernible). Sample 1 NA, however, exhibits a better pore arrangement than sample 2 NA according to the higher intensity and smaller width of the (100) reflection. It is known that the pore order is dictated by the degree of surfactant packing during silica condensation which is clearly influenced by varying the acid content. While both products of 1 NA and 2 NA have the typical mesoporous type IV sorption isotherms, sample 1 NA exhibits two broad capillary condensation steps: one with no hysteresis loop occurring at 0.2 to 0.35 p/p_o_ and one at 0.4 to 0.7 p/p_o_ with type H2 hysteresis loop. This indicates the presence of intraparticle and interparticle porosities in sample 1 NA which result in a bimodal pore size distribution having average sizes of 2.5 and 3.8 nm. The interparticle porosity emerges possibly from the aggregation of small particles during condensation. Sample 2 NA conversely has an average pore size of 2.9 nm. Pore size and area properties are shown in Table 2.

The above results suggest that pore structure becomes more arranged at lower nitric acid molar ratios. Synthesis at 0.2 and 0.5 NA molar ratios confirms this observation where the sharper (100) reflections plus additional high reflection peaks characteristic of a hexagonal pore arrangement become visible at 3° to 4° 2*θ* of the XRD pattern (Figure 7a). Nitrogen sorption isotherms of these samples in Figure 6b show type IV isotherms. Unlike the MSF sample, capillary condensation of sample 0.2 NA extends over a wider p/p_o_ range and shows type H2 hysteresis loop (sloping adsorption and vertical desorption). This suggests that pores in the 0.2 NA spheres have narrow and wide sections and possible interconnecting channels [44]. Conversely, capillary condensation step of sample 0.5 NA was sharper, which is indicative of a uniform pore size as verified by its more resolved XRD peaks. Surface area properties of these two samples are very close (Table 2). Noteworthy is their pore size (approximately 3 nm) which is slightly larger than the MSF (2.35 nm), suggesting that NO_3_^−^ counterion causes swelling in the surfactant micellar size. Similarly, the larger wall thickness (2.3 to 2.45 nm vs. 1.95 nm for MSF) means more silica condensation in these samples. However, they have smaller surface areas (624 and 560 vs. 1,008 m^2^/g) and pore volumes (0.43 and 0.4 vs. 0.64 m^3^/g). Overall, high nitric acid concentrations provide spheres with uniform pore size and disordered structure, whereas growth at low concentrations increases the rate of condensation and surface roughness and promotes pore order.

Quiescent preparations using sulfuric acid were slightly different. The rate of silica production was slower for H_2_SO_4_ than HCl or HNO_3_ due to weak binding of the SO_4_^−2^ counterion to CTA^+^ surfactant according to the Hofmeister series [45]. This reduces the condensation rate and delays precipitation of products to a period exceeding 2 weeks. Preparations conducted at 1 SA and 2 SA molar ratios gave essentially similar results. The output mix of morphologies in Figure 5 has disordered hexagonal pores. According to the XRD pattern in Figure 7a, they show only a broad (100) peak. Sorption isotherms are also of type IV but with a slightly wider capillary condensation step. The average pore size is about 2.5 nm, which is very close to the pore size of MSF, but the wall thickness is thinner (approximately 0.8 vs. 2.0 nm for HCl growth and 2.15 nm for HNO_3_ growth), emphasizing our point of slow condensation in the presence of H_2_SO_4_ acid which becomes even slower at higher molar ratios (3.34 SA), where no silica was observed in the growth beaker.

In line with the above results, quiescent interfacial growth is a slow process (>2 days) and can be influenced by the counterion type and content. At equivalent acid contents, the growth time increased in the order of NO_3_^−^ < Cl^−^ < SO_4_^−2^. This aligns with the known Hofmeister series of anions’ binding strengths to cationic surfactants which decrease in the order of NO_3_^−^ > Cl^−^ > SO_4_^−2^[45,46]. This means that the highly binding NO_3_^−^ counterions can associate easily to surfactant micelles (S^+^) and shield the positive charge forming S^+^X^−^ associates with a higher apparent negative charge in the water phase. Accordingly, the attraction rate to the positive silica species (I^+^), which have already diffused into the water phase and hydrolyzed with water, will increase and lead to faster silica condensation and shorter induction times. With a less binding counterion, like Cl^−^, the S^+^X^−^ species become less negative which reduces the attraction to (I^+^) and increases the induction time. In the case of the weakly binding SO_4_^−2^ counterion, only slight proportions of this counterion can be associated, thus keeping a strong repulsion between the similarly charged surfactant and silica species. This hinders the condensation process and slows the growth as seen in sample 3.34 SA.

The condensation of silica continues on the silica-surfactant seeds in the water phase, and further steps of aggregation and restructuring can simultaneously take place which in summary control the morphology and pore structure of the final product. It should be noted that at a fixed amount of CTAB surfactant, the variation of acid content basically changes the excess amount of acid in the water phase which influences the physical chemistry of growth. Among these influences are solvent evaporation and surfactant packing. Seshadri et al. have recently reported that increased evaporation of water and alcohol at the interface is a key parameter for changing local concentrations and the degree of surfactant packing in interfacial growth [47]. The inferior pore order observed at high nitric acid contents and with sulfuric acid can be attributed to this phenomenon. SO_4_^−2^ anion has a large size and can bond weakly to more water molecules than NO_3_^−^. Similarly, at high nitric acid content, excess NO_3_^−^ ions will bind to water molecules and reduce their tendency to evaporate. This causes localized dilution and loose packing of surfactant species within the water phase which leads to the observed low order/disordered structures (TEM Figure 4a and XRD Figure 7a). Similarly, localized dilution slows silica condensation which emerges as spherical morphologies (Figure 4a). More corrugation and better order were the case at low acid contents due to more evaporation which causes more packing, higher local concentrations, and faster silica condensation (Figures 4e and 7a).

### Effect of silica source

**Figure 8:**
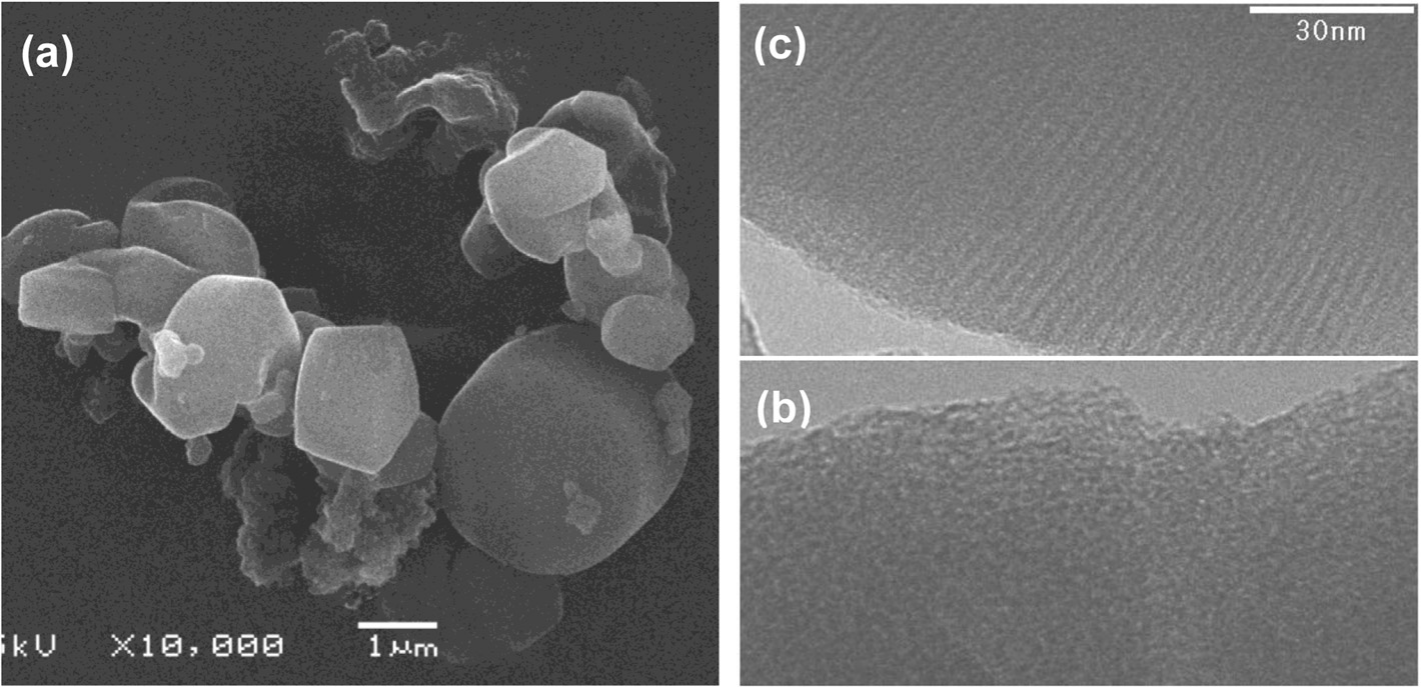
**SEM (a) and TEM (b**, **c) images of sample MS4 prepared using TESO and HCl.**

Effect of the silica source on the quiescent growth product is represented by sample MS4 in which TEOS substituted TBOS while keeping all other conditions unchanged. TEOS is less hydrophobic than TBOS, so it can diffuse more easily into the water phase and condense in the presence of surfactant micelles into mesoporous silica. The translucent water phase solution took a shorter period (a few hours) than the TBOS precursor (approximately 2 days) to form a turbid solution of fine suspended solids plus a layer at the interface. The layer got thicker with time and was accompanied by growth and precipitation of fine white particles in the water bulk. Unlike TBOS, no fibers were seen at the interface with TEOS. TEOS alters the fiber formation mechanism and leads to nonfibrous shapes as confirmed by the SEM image in Figure 8a. Silica collected from the fine precipitate in the water phase bulk consists of twisted particles and gyroidal shapes having a wide and shallow (100) XRD peak in the low 2*θ* range (Figure 7b). This peak is characteristic of a mesopore system lacking the long-range order similar to the structure obtained in the presence of nitric acid (3.34 NA) and sulfuric acid.

Nitrogen sorption isotherms of the TEOS-based product and the corresponding surface area properties are given in Figure 6a and Table 2. Type IV isotherms were obtained with a broad capillary condensation step, pointing out the presence of a wide pore size distribution. The distribution of pore sizes (not provided here) shows that the majority of pores are smaller than 4 nm, and some pores, to a lesser extent, are wider than 5 nm. The average pore size is 3.7 nm (larger than the 2.35-nm size of TBOS-based silica fibers), and surface area is 475 m^2^/g. In view of these outcomes, self-assembly using TEOS in quiescent conditions yields a mesoporous structure with disordered pore arrangement as verified by TEM imaging (Figure 8b). Spots possessing long nonconnecting channel that resulted from wormlike micelles can be observed (Figure 8c). TEOS in the presence of Cl^−^ counterion causes elongation of the short cylindrical micelles of the surfactant into long wormlike micellar templates. However, this combination does not induce ordering of these micelles upon silica condensation.

**Figure 9:**
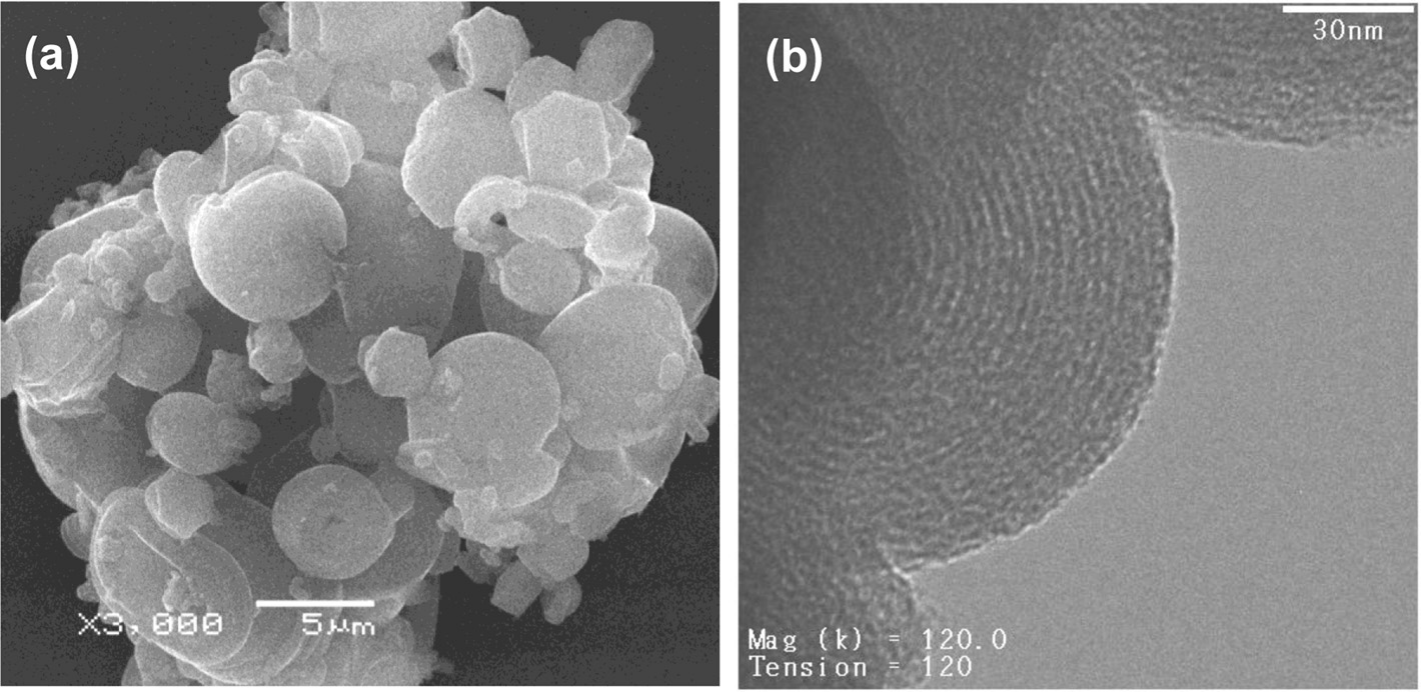
**SEM (a) and TEM (b) images of sample MS6b prepared using TEOS and HNO**_**3**_**.**

A similar morphology was obtained for the quiescent condensation of TEOS in the presence of HNO_3_ (sample MS6b). The gyroidal product (Figure 9a) possesses a slightly better pore arrangement, indicated by the sharper (100) reflection in the XRD pattern (Figure 7b), but has inferior surface area properties (Table 2). In mesoporous structure growth, it is known that the self-assembled silica-micelles species undergo further condensation and structuring (pore ordering) steps that dictate the final shape and structure. The better order can be related to a better packing of surfactant micelles under nitric acid compared to HCl which goes in line with the Hofmeister binding strength, NO_3_^−^ > Cl^−^, so there are more attraction and formation of self-assembled species. However, subsequent restructuring was slower for HNO_3_ than for HCl as indicated by inferior structural properties (smaller pore width and surface area). Long wormlike pores are still seen in the TEM image (Figure 9b) and apparently extend over the curvature and surface texture of the product. The repetition of this structure, regardless of the acid type, stresses the role of TEOS in elongating the wormlike micelles under quiescent conditions. It is known in mixed systems that cationic surfactants can grow long under some conditions favoring the reduction of end-cap energy of the rod micelles [48,49].

The general behavior is that TEOS under quiescent conditions yields mesoporous gyroidal shapes in the water bulk with lower pore order and structure quality than TBOS. The key difference lies in the speed of condensation and the simultaneous pore structuring steps. As described before, TEOS is less hydrophobic, so it can diffuse from the top layer into the water phase faster than TBOS. This was clearly reflected by the shorter induction time. Thus, in the absence of mixing, TEOS can be available more readily in the water phase than TBOS and hence speeds up the condensation, yielding products mostly in the bulk of water phase. Particle aggregation was noticed but not in well-defined shapes. Simultaneous pore structuring was ineffective or even absent as reflected by the lower degree of order. In addition, high local concentration of TEOS under highly acidic conditions offers opportunity to silica condensation without involvement of surfactant leading to amorphous particles which reduces the overall order generally depicted by XRD as poor patterns.

The gyroidal morphology of TEOS growth resembles the outcomes in well-mixed systems. TEOS changes the growth behavior and alters the linear formation of fibers observed with TBOS. The slow diffusion of the TBOS species at the interface balanced with proper speed of condensation and restructuring causes their immediate consumption in the water phase at the interfacial region and yields seeds that grow linearly into fiber shapes [37]. In a recent work, we demonstrated that mixing the water phase during TBOS diffusion changes the linear growth and yields three-dimensional (3D) gyroidal shapes [47]. A similar morphology was seen quiescently using TEOS. This confirms that the fast diffusion of the TEOS species makes them available in the water phase homogenously where they condense with surfactant seeds into three-dimensional particles. These particles undergo further condensation and aggregation to form the final gyroidal shapes, but pore restructuring is not sufficient to improve the pore order.

### Effect of surfactant type

The effect of surfactant was investigated by replacing the cationic CTAB surfactant with the nonionic Tween surfactant. Two different hydrophobic alkyl chain lengths were used: monolaurate (Tween 20, coded T20, *R* = C_11_H_23_) and monooleate (Tween 80, coded T80, *R* = unsaturated C_17_H_33_); T 80 being more hydrophobic. As suggested by several investigators, the species interact via the (S^0^H^+^)(X^−^I^+^) route under acidic medium where S, I, and X are the organic micelles, inorganic species, and halide anion, respectively. In this set, we used the TEOS silica precursor instead of the TBOS to facilitate comparison with the reported Tween-TEOS products assembled under mixing conditions [50-53].

After a few hours of induction time, the clear-water phase turned turbid to an extent that is inversely proportional to surfactant hydrophobicity (turbidity T20 > T80). For T20, a cotton-like network of silica appeared by day 2 and spread out to fill the water phase by the fourth day. The network remained suspended in the water phase throughout the growth time. Loose particle precipitation was also seen in the water medium. For T80, the trend was different. The water phase turned from turbid to milky and remained like that over the remaining time. For both surfactants, a progressively thickening film of silica was visible at the interface, part of which precipitates with time into the water phase. If the solution is left for prolonged periods (>20 days), more notably with T80, the excess surfactant will yield an oily layer, mediating the silica film and milky solution. For synthesis with TBOS, the growth becomes slower (longer induction time) and the cotton-like network can be visible for both T20 and T80 surfactants.

**Figure 10:**
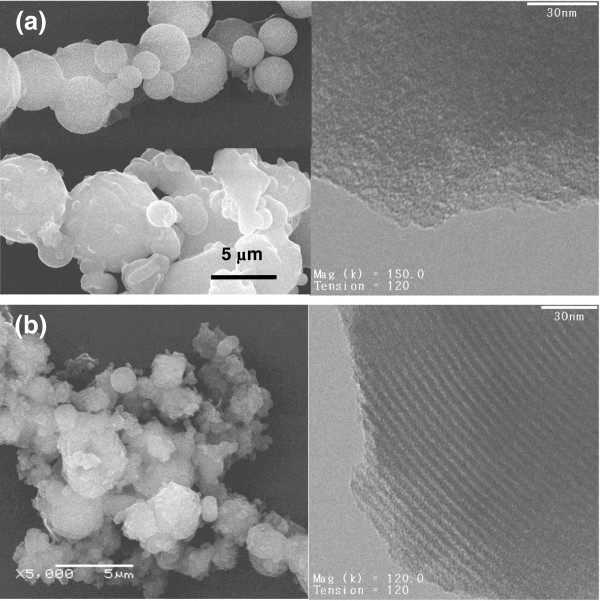
**SEM (left) and TEM (right) images of samples prepared using nonionic surfactants. (a)** MS5a using Tween 20 and **(b)** MS5b using Tween 80.

SEM images showing the particle texture of the Tween-based silica are displayed in Figure 10. The product of T20 consists of smooth and nonuniform spheres. No real fibers or linear shapes were seen in the images, suggesting that the cotton-like bundles observed in the growth medium were basically loose particle agglomerates. Surface corrugation and nonuniform shapes develop as a result of irregular condensation. With T80 surfactant, the output is mostly ill-shaped agglomerates of preformed spheres that cause combined intra- and interparticle textures. Part of the irregular shapes is contributed by precipitation from the thick film grown at the interface. This film was shown in an earlier study to be amorphous with low surface area properties [37].

According to N_2_ sorption isotherms (Figure 6a), the Tween-based products have mesoporous structures with a shallow capillary condensation step indicating a nonuniformity in pore sizes. As seen in Table 2, the average pore size for the T20 product is 3.0 nm which is larger than both the TEOS-based gyroids (MS6b, 2.64 nm) and TBOS-based fibers (MSF, 2.35 nm) but has surface area and pore volume properties inferior to the MSF product. An additional capillary condensation step at p/p_0_ = 1 was seen for the T80 product as a result of the textural porosity generated from the interparticle spaces in the random agglomerates observed in the SEM image (Figure 10b). This shifts the average pore size to a higher value (3.7 nm), combining the structural intraparticle mesopores and the larger size textural interparticle pores. Such interparticle spaces were not seen in the T20 product because the particles of T80 silica are smaller and aggregated and would therefore provide an additional textural porosity.

The XRD patterns of Tween-based silica in Figure 7b show poorly ordered structures (MS5a and MS5b). The T20 silica shows an amorphous response without any peak reflection, while the T80 product exhibits a single broad diffraction peak characteristic of a mesopore system lacking enough order. This structure was further confirmed by TEM images. Figure 10a clearly shows that T20 silica has irregular porous regions characteristic of an amorphous structure. Conversely, the T80, which showed a small reflection in the XRD pattern, displays some domains of ordered assemblies appearing as long wormhole-like channels along the *c*-axis (Figure 10b). These results suggest that acidic interfacial growth with neutral surfactants produces mesoporous structure with poor channel arrangement. This structure is similar to MSU-X materials prepared with Tween surfactant by the S^0^I^0^ route under neutral and mixing conditions [50]. It is interesting to note that silica prepared with TEOS-T80 system (sample MS5b) has properties very close to the TEOS-CTAB system (sample MS4); both have poor order and wormlike mesopores. They only differ in morphology.

The main reason behind the poor order in neutral surfactants is the weak (S^0^H^+^)(X^−^I^+^) interaction which becomes even worse in the absence of mixing. This weak attraction of silica-surfactant seeds plus the slow structuring step associated with quiescent growth are unfavorable for pore ordering. Enhancement of structural order in the (S^0^H^+^)(X^−^I^+^) route of MSU-type silica was achieved in earlier studies by operating at a surfactant concentration higher than 16 wt% in acidic conditions (pH <2) [54] or by addition of a fluoride mineralizing agent (e.g., NaF) at neutral [50] or pH >2 conditions [55]. Our system achieved the mesostructure at 0.7 wt% surfactant concentration, so we believe that ordering can be improved in quiescent interfacial growth by the addition of a structure-enhancing agent.

### Mechanism of quiescent interfacial growth

The above studies indicate that the quiescent interfacial approach for acidic synthesis of mesoporous silica is sensitive to growth parameters. TBOS or TEOS placed as a top layer diffuses through the stagnant interface, hydrolyzes with water, and then condenses with surfactant seeds in the water. Similar to the colloidal phase separation mechanism in mixed systems [31], silica-surfactant composites in quiescent growth phase-separate and undergo further condensation, pore restructuring, and aggregation steps. Interrelation among these simultaneous steps, driven by the growth conditions, is not clear in quiescent approach, but they clearly dictate the final shape and structure. The product develops slowly into rich textural morphologies composing mainly of fibers attached to the interface and/or particulate shapes in the water bulk. These shapes possess wormlike mesochannels of uniform size and pore arrangement ranging from poorly ordered (particulates) to well-ordered p6mm-type hexagonal structures (fibers). The external morphology and internal structure vary with the type and content of the silica precursor, acid source (counterion), and surfactant type.

The slow growth nature of the quiescent approach (order of days) is attributed to the absence of mixing plus the slow interdiffusion among the hydrophobic (TEOS/TBOS)-hydrophilic (water) constituents. Silica source diffuses slowly from the top layer into the water causing a distribution of silica concentration in the stagnant water bulk. This distribution can drive the condensation faster or slower. Moreover, the distribution is highly influenced by solvent concentration (water + alcohol) in the water phase driven by their tendency to evaporate at the interface [56]. By removing the solvent from the interface upon hydrolysis, surfactant seeds become more closely packed which enhances the structural order. Similarly, evaporation brings uncondensed silica species in contact which drives the system into faster condensation. Thus, the rate of silica diffusion and solvent evaporation are key determinants of shape and structure in the quiescent approach. This is due to the fact that they cause a variation in the local concentrations across the water phase from interface to bulk which leads to relative differences in the products grown across this phase.

**Figure 11:**
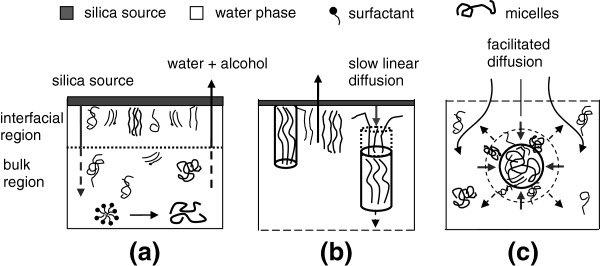
**A schematic representation of the quiescent interfacial**-**bulk growth mechanism. (a)** Initial two-phase configuration and the suggested interfacial and bulk regions, **(b)** interfacial region where slow linear supply of silica source in packed micelles yields linear growth of ordered silica fibers, and **(c)** bulk region where facilitated silica diffusion to loosely packed micelles yields 3D growth of low-ordered spheres and gyroids.

In this regard, on combining the results of our study, we can imagine the water phase in the quiescent medium to be composed of two regions: an ‘interfacial region’ existing just below the silica source-water interface and a ‘bulk region’ comprising the remaining water bulk phase located below the interfacial region. The growth behavior in each region is unique as a result of variations in reactant availability and local concentration. A schematic representing the proposed growth process in each region is given in Figure 11. Surfactant molecules originally present in the water phase assemble into rod and wormlike micelles during the premixing of the acidic water medium (Figure 11a). Silica species start to diffuse slowly through the interface and undergo hydrolysis with water forming an amorphous film at the micelle-free interface. Due to the absence of mixing, slow diffusion makes the hydrophobic silica precursor initially present in the interfacial region. However, some experimental factors were noticed to shift silica condensation to the bulk region by facilitating the diffusion of the silica species into that region. These factors are the acid type, hydrophilicity of silica source, and surfactant involved. In the interfacial region, the diffusing species assemble with surfactant micelles forming silica-surfactant seeds that can grow by the addition of more silica and surfactant species.

In the TBOS studies with HCl (sample MSF), growth was restricted to the interfacial region where the seeds begin to grow by the addition of more silica and micelles at the interface. Silica species were consumed instantly by the seeds at the interface. The slow supply and instant consumption of TBOS was seen as a linear diffusion, and the seeds grow likewise into linear fibrous shapes [37] as shown in Figure 11b. The fibers have a highly ordered hexagonal structure. One aspect of this order is evaporation at the interface. Due to solvent evaporation, both surfactant micelles and uncondensed silica-surfactant seeds are closely packed (higher local concentrations) which enhances condensation and promotes restructuring of the pores. It is also known that pores can restructure as long as the condensation is not complete. The longer the growth time, the better is the order of end product grown in the interfacial region [37]. In comparison, the bulk region is highly diluted by the solvent. Thus, micelles and condensed specie are less packed; therefore, condensation and pore restructuring are relatively slower over there and lead to less ordered structures.

On replacing HCl with HNO_3_, where NO_3_^−^ is more binding, the growth shifts to the bulk phase (sample MS7) driven by facilitated diffusion because the more negatively charged S^+^NO_3_^−^ micelles attract TBOS more than the S^+^Cl^−^ micelles. This is believed to shift the condensation of silica towards the bulk phase. Hence, TBOS in this diluted region gets supplied to the less packed micelles from all sides, causing the slow condensation of uncondensed species into three-dimensional shapes including smooth and corrugated spheres with poor order (Figure 11c). Unordered pore structure, observed while increasing HNO_3_ content, can be partly assigned to the evaporation tendency. The extra counterions can hydrogen-bond to water molecules and hinder their evaporation, which reduces the local concentration and packing of the surfactant.

Similarly, the use of TEOS causes facilitated diffusion of silica source into the bulk region because it is more hydrophilic than the TBOS. This facilitated diffusion accelerates the spread of TEOS in the water phase. Unlike the unidirectional supply of TBOS, TEOS becomes supplied from all directions, causing the growth of 3D particulate gyroidal shapes to be much like those prepared under mixing conditions. They have poor structure reflected by the loose micellar packing in the bulk region. In earlier quiescent interfacial studies, fibers were prepared from TEOS by dissolving it in a hydrophobic solvent (e.g., hexane) [32,36]. This reduces the diffusion of TEOS and gives linear supply and linear shapes in agreement with our suggestion of slow vs. facilitated diffusion. We have recently demonstrated that mixing of the water phase while quiescent interfacial growth using TBOS alters the linear supply of TBOS and leads to gyroidal shapes [47]. When employing a neutral surfactant, growth shifts to the bulk region both for TBOS and TEOS sources. It is not well understood why growth becomes faster than the ionic surfactants (CTAB), but the simultaneous effect of low binding of S^0^H^+^X^−^I^+^ and the fast condensation (driven by facilitated diffusion and low pH) ends up with irregular shapes of disordered structures.

There is one final note about the morphology and pore structure. Evaporation and facilitated diffusion in the proposed interfacial-bulk mechanism under a highly acidic medium (pH <1) causes a variation in the rate of condensation. Because of nonmixing, condensation becomes generally slow, but it is relatively faster in the interfacial region than in the bulk. It is also known that pore restructuring and aggregation act simultaneously along condensation in acidic growth. The relative rates of these steps define the final shape and structure [46]. Growth with NO_3_ gives smooth spheres in the bulk region, and this shape is typical to slow condensation to minimize surface tension [31,57]. Use of TEOS, on the other hand, increases the rate of condensation and gives twisted surfaces and gyroids to minimize surface tension. However, in all cases, pore restructuring was slow compared with condensation and aggregation steps unless the growth is maintained in the interfacial region.

An ultimate goal of any self-assembly method is the ability to control the particle size and shape effectively while achieving high pore uniformity. Such output is possible in mixed systems where a number of uniform morphologies have been demonstrated. In quiescent systems, on the other hand, effective control of the size and shape is still unattainable with high fidelity due to the progressive nature of silica diffusion which varies the location and speed of growth. The ability to restrict the growth in a selected region by manipulating the additives would result in a better control of the product uniformity. This is similar to producing fibers at the interface or spheres in the bulk exclusively. However, more work is needed to improve the pore uniformity of the outputs. Future research on this approach should address factors to enhance pore restructuring such as the addition of mineralizing agents.

## Conclusions

Variation of the silica source, acid type and content, and/or surfactant type leads to important changes in the acidic self-assembly of mesoporous silica under quiescent interfacial conditions. TBOS combined with HCl-CTAB provides a tight balance of slow diffusion and condensation/restructuring processes for the formation of silica fibers with high order in the interfacial region. The use of a more binding acid (e.g., HNO_3_), a more hydrophobic silica source (TBOS), or a neutral surfactant disturbs this balance and shifts silica diffusion into the bulk, causing 3D growth of particulates with poor structural order. The combined effect of slow silica source diffusion and water-alcohol evaporation at the interface is postulated to cause variation in the local silica and surfactant concentrations among the interfacial vs. bulk regions and hence in the shape and order of the product. Enhancement of pore restructuring is an important issue to address in future studies of quiescent interfacial approach.

## Abbreviations

BET: Brunauer-Emmett-Teller; BJH: Barret-Joyner-Halenda; CTAB: Cyteltrimethlammonium bromide; MSF: Mesoporous silica fibers; SEM: Scanning electron microscopy; TBOS: Tetrabutyl orthosilicate; TEM: Transmission electron microscopy; TEOS: Tetraethyl orthosilicate; Tween 20: Polyethylene oxide sorbitan monolaurate; Tween 80: Polyethylene oxide sorbitan monooleate; XRD: X-ray diffraction.

## Competing interests

The authors declare that they have no competing interests.

## Authors’ contributions

HMA carried out the main experimental work and drafted the manuscript. AA conducted part of the experiments under the supervision of HMA and MAA. MAA participated in the sample characterization and analysis. JYSL participated in the discussion of results and helped make critical comments in the initial draft of the manuscript. All authors read and approved the final manuscript.

## Authors’ information

HMA is an assistant professor at The University of Jordan. MAA is an assistant professor at German-Jordanian University. AA was a research assistant at German-Jordanian University and is currently an MSc student at Masdar Institute of Science and Technology, United Arab Emirates. JYSL is a professor at Arizona State University.
